# The Surgical Aid Society

**Published:** 1902-12-13

**Authors:** 


### THE SURGICAL AID SOCIETY.

### ENCOURAGING STATEMENTS.

The 40th annual meeting of the Surgical Aid Society took place at Cannon Street Hotel on Monday afternoon (December 8th), when the Lord Mayor, Sir Marcus Samuel, was in the chair, and with him at the table were Alderman and Sheriff Sir G. W. Truscott, Sheriff Sir T. H. Brook-Hitching, the Ven. Archdeacon W. M. Sinclair, D.D., the Very Rev. Hermann Adler, Ph.D., LL.D. (Chief Rabbi), and others.

The business of the meeting was to receive the annual report and accounts and to pass one or two resolutions, and the whole did not occupy more than 40 minutes. Letters of apology had been received from Sir Horace Brooks Marshall, who enclosed 10 guineas; Mr. J. Herbert Tritton (5 guineas enclosed); Lord Rothschild, the Bishop of London, the Bishop of Stepney, who regretted his inability to be present, all the more because he had seen the valuable work done by the Society so often and in so many places; the Dean of Westminster, the Mayor of Croydon, Mr. L. Cohen (enclosing 5 guineas), Judge Bompas, and the secretaries of Bethnal Green and Colchester branches.

The Lord Mayor's Speech.

The report and accounts were taken as read, and their adoption was moved from the Chair. The value and worth of an institution, said the Lord Mayor, were proved by the demand that there was for the objects it was founded to supply, and he could safely say that, though belonging to a great many societies, he received the greatest number of applications for help as a subscriber to this society and one other (the Earlswood Asylum). This showed that the societies in question were supplying a need that was abundantly manifest. He had sometimes feared that a certain amount of bartering and selling of tickets went on, but he was sure that this was impracticable. He always tried to make it so himself by filling in the full particulars on the letter before parting with it. If all subscribers would do this, traffic in tickets would be avoided. Full precautions were taken by the Society in requiring the surgeon's certificate in each case before the appliance could be ordered, and this did away with all possible objection to a society of this kind. The suffering was caused in almost all cases by circumstances over which the patients had no control, and to relieve it was a very laudable work.

### Increase of Income.

Archdeacon Sinclair then ran over the chief points in the report. He was sure that all who had turned out on such an inclement afternoon must be genuinely anxious to relieve the maimed, halt, and blind. The committee were wise in holding the meeting some days before Christmas, because people's hearts were stirred to sympathy at such a time. He rejoiced that the income of the Society had been better in spite of all the drains on the charity of the public. Last year there was a donation of £1,000; this has not been repeated, but apart from this, there was a virtual increase of £868 in donations and £807 in annual subscriptions. The local branches had sent in £170 more. Legacies amounted to £1,954 18s. 9d. All this implied, of course, an increase in expenditure, *i.e.*, by £1,400, of which £1,300 was spent in surgical appliances. Twenty-two of the City companies had subscribed, and the Bethnal Green Working Men's Benevolent Society had sent in £189. This showed the estimation in which the Society was held by the class it helped to relieve.

### Two Thousand Additional Cases Helped.

The very large number of 18,709 patients had been helped, more than 12,000 of whom were women and children; and the committee recorded with great satisfaction that upwards of a quarter of a million people had been benefited during the 40 years the Society had been in existence. The number of appliances was, of course, greater than last year—29,895 altogether, being an increase of 2,008. The loan department had been instrumental in supplying 388 water and air beds, carriages, crutches, etc. He thought that a very useful arrangement which enabled subscribers who had no immediate use for their letters to return them to the secretary; 1,665 had been used in this way, for 593 special cases. The Mackeson Fund had helped 91 of the very worst cases. Owing to the increase of work the committee had thought it desirable to supply daily and weekly papers in the waiting-room, in order that the patients' time might not be wholly wasted. The Society deeply regretted the death of Mr. William Gray, at the advanced age of 80 years; he had known the Society from the beginning, and his practical interest was maintained to the last. They also greatly felt the loss of Mr. W. Nicholson, chief clerk and collector, who, after 29 years of faithful service, died on February 14. The speaker concluded by saying tha with the increase of population there was also an increase in cases requiring surgical treatment; he believed there were more street accidents in one year than in a costly and sanguinary campaign, and the satisfaction arising from money spent on costly amusements, dresses, wines, entertainments and pictures could not be compared with the delight of enabling a bread-winner to return to his employment after temporary disablement.

### Appointments.

The report and accounts having been adopted, Dr. Adler (the Chief Rabbi) proposed that the vacancy caused by the death of Mr. Gray should be filled by the appointment of Mr. Samuel Watson (who for many years had rendered valuable service as a member of committee and a Trustee) as Treasurer and Chairman of Committee. Mr. Watson's father was one of the founders of the society, and it would be admitted by all who had worked with him that no one had shown more zeal or energy than Mr. Samuel Watson.

The Rev. J. G. Pilkington having seconded the proposal, it was carried unanimously, and Mr. Watson said he would do everything within his power to justify the appointment.

Mr. John Robert Cooper, J.P., D.L., had accepted the trusteeship vacated by Mr. Watson, and the vacancy on the committee caused by this change had been filled by the election of Mr. Benjamin Densham, of Bramley Croft, Croydon, who has long been an ardent and generous supporter of the Society. These appointments were confirmed by the meeting.

### One of the Best Societies in the City.

A vote of thanks to the surgeons, Mr. F. R. Fisher, F.R.C.S.; Mr. E. Muirhead Little, F.R.C.S.; and Mr. T. M. Openshaw, C.M.G., M.S., F.R.C.S., was carried enthusiastically, and the usual thanks to the chair followed. In his reply, the Lord Mayor said those who imagined that merchants went to the City only to make money did them a very great wrong. Many were exceedingly generous in helping philanthropic objects. He had no hesitation in saying that in the future, as in the past, the Lord Mayors of London would be glad to help the Surgical Aid, which was one of the best societies in the City.

